# Survival from multiple myeloma in England and Wales up to 2001

**DOI:** 10.1038/sj.bjc.6604607

**Published:** 2008-09-23

**Authors:** B Rachet, E Mitry, A Shah, N Cooper, M P Coleman

**Affiliations:** 1 Cancer Research UK Cancer Survival Group, Non-Communicable Disease Epidemiology Unit, Department of Epidemiology and Population Health, London School of Hygiene and Tropical Medicine, Keppel Street, London WC1E 7HT, UK; 2 Département d'Hépatogastroentérologie et Oncologie Digestive, Centre Hospitalo-Universitaire Ambroise-Paré, 9 avenue Charles de Gaulle, Boulogne F-92100, France; 3 Social and Health Analysis and Reporting Division, Office for National Statistics (Room FG/114), 1 Myddelton Street, London EC1R 1UW, UK

Multiple myeloma is an uncommon neoplasm of plasma cells affecting mainly the elderly; it is rare under the age of 50 years. Each year in England and Wales, approximately 1800 cases are registered in men (1.5% of all malignancies) and 1500 in women (1.2% of all malignancies). Incidence has increased steadily since the 1980s in both sexes in many countries, including England and Wales (Coleman *et al*, 1993; Quinn *et al*, 2001). Incidence is slightly higher in men, and it has risen by approximately 12% in both sexes since the mid-1980s to reach 6.1 per 100 000 per year in men and 5.5 in women by 1999. No marked socioeconomic gradient in incidence has previously been reported in England and Wales, but the most deprived fifth of the population provides a striking exception to the overall trend in the 1990s: incidence rates in this group increased very little in either sex, and by 1999, annual incidence was 20–25% lower than in the other four deprivation groups (4.5 and 4 per 100 000 in men and women, respectively).

The aetiology of myeloma is unknown, although exposure to ionising radiation is a cause (IARC, 2000) and occupational exposure to chemicals, such as ethylene oxide, styrene and vinyl chloride have been suggested as risk factors (Blair and Kazerouni, 1997; Lynge *et al*, 1997; Stellman, 1998).

Myeloma is characterised by a malignant clone of plasma cells invading bone marrow, with high serum or urine levels of monoclonal immunoglobulin (usually a *γ*-globulin), and lytic bone lesions. It is often preceded by a latent monoclonal gammopathy of uncertain or undetermined clinical significance, which may last for years before evolving into malignant myeloma proper. Monoclonal gammopathy of uncertain or undetermined clinical significance may be diagnosed incidentally from routine examination of serum or a blood film, and in the minority of patients who do go on to develop myeloma, the date of diagnosis – and thus the recorded duration of survival – will depend on the frequency and diagnostic intensity of clinical follow-up (Berrino *et al*, 1995). Part of the increase in incidence may be attributable to the improved sensitivity of diagnostic tests, but environmental exposure to toxins may also underlie a real increase (Richardson *et al*, 2004).

Myeloma comprised 96% of the malignant monoclonal gammopathies throughout the period 1986–1999, whereas plasmacytoma accounted for most of the remainder.

Almost 40 000 adults were registered with multiple myeloma in England and Wales during the 14-year period 1986–1999. Approximately 18% of patients otherwise eligible for survival analysis were excluded, mostly (13%) because their recorded duration of survival was zero (date of diagnosis same as the date of death). Some of these patients will have been diagnosed on the day of death, but in many cases the cancer registration was based solely on the death certificate; hence, the date of diagnosis was unknown. It was not possible to distinguish these cases in the available data and all patients with ‘zero survival’ were thus excluded. The vital status of 1.6% of eligible patients was unknown on 5 November 2002, when the data were extracted for analysis, and a further 3% were excluded because myeloma was not their first primary malignancy. The distribution of patients in these categories was fairly stable over the period 1986–1999 and similar in all deprivation groups (data not shown); hence, the exclusions are unlikely to have had a large impact on time trends or the deprivation gradient in survival.

## Survival trends

**Figure 1:**
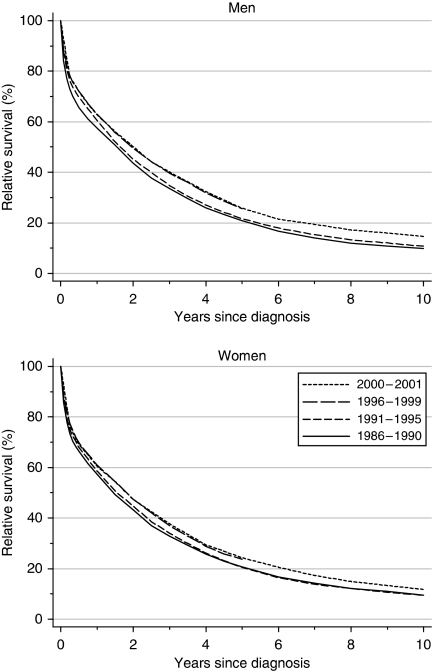
Relative survival (%) up to 10 years after diagnosis by sex and calendar period of diagnosis: England and Wales, adults (15–99 years) diagnosed during 1986–1999 and followed up to 2001. Survival estimated with cohort or complete approach (1986–1990, 1991–1995, 1996–1999) or hybrid approach (2000–2001) (see Rachet *et al*, 2008).

For patients diagnosed during 1996–1999, relative survival was approximately 62% at 1 year and 25% at 5 years, whereas 10-year survival was only 10--11% for patients diagnosed during the early 1990s (Table 1). Survival is now 1–2% higher in men, but the differences are not significant. After adjustment for changes in the distribution of patients by deprivation category, 5-year survival is seen to have risen significantly over the 14-year period 1986–1999, by an average of approximately 5% every 5 years (4.4% in men, 5.8% in women). In fact, most of the increase occurred during the 1990s (Figure 1). One-year survival also increased significantly for men (3.8% every 5 years) to 62.9% (Table 1, Figure 1), but the small increase for women was not significant. There was no significant increase in 10-year survival between patients diagnosed in the late 1980s and those diagnosed in the early 1990s.

Hybrid analysis (Brenner and Rachet, 2004) suggests that for patients diagnosed during 2000–2001, the national average relative survival up to 5 years should remain stable (Table 1).

## Deprivation

One-year survival has been consistently and significantly higher for more affluent men. The deprivation gap widened from −5 to −8% during the period 1986–1999 (Table 2), although the average change in the gap every 5 years (−1.2%) was not itself statistically significant. For women, the deprivation gap in 1-year survival is not significant.

**Figure 2:**
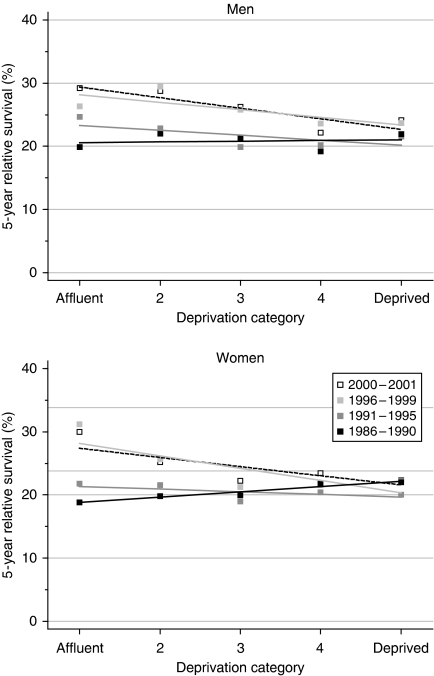
Trends in the deprivation gap in 5-year relative survival (%) by sex and calendar period of diagnosis: England and Wales, adults (15–99 years) diagnosed during 1986–1999 and followed up to 2001.

There was no significant difference in 5-year survival among deprivation categories for men diagnosed during 1986–1990. The deprivation gap of +3.4% in 5-year survival among women diagnosed at that time (higher survival in more deprived women) is unusual, but it was of borderline statistical significance (Table 2), and the data are well fitted by a simple linear regression (Figure 2). Furthermore, the positive deprivation gap in 10-year survival for women diagnosed during the late 1980s (+4.6%, higher survival in the most deprived) was statistically significant at the 1% level.

During the 1990s, however, the more typical deprivation pattern in 5-year survival emerged between both sexes (negative gradient; lower survival in the more deprived groups). The deprivation gap of −5% that has arisen for men diagnosed in the late 1990s is of borderline significance. For women, the positive deprivation gradient in myeloma survival (+3%, higher survival in more deprived women) in the late 1980s had completely reversed by the late 1990s: by then, the deprivation gap between the most deprived and the most affluent women (almost −8%, lower in more deprived women) had become statistically significant at 5%. The rate at which the deprivation gap in 5-year survival for women has widened (−5.6% every 5 years) is itself significant (Table 2). Figure 2 illustrates these wide differences among deprivation groups in survival trends, with no improvement at all in 5-year survival among the most deprived group, but an increase of more than 10% for the most affluent group, in which survival has now overtaken that in the most deprived group.

Short-term predictions with hybrid analysis suggest that the wide socioeconomic differences in survival are not likely to reduce in the near future (Table 2).

## Comment

Despite substantial overall gains in survival from multiple myeloma, both short-term and long-term survival remains poor. Even for patients diagnosed 2000–2001, survival is predicted to be below 30% at 5 years, and below 15% at 10 years after diagnosis. The improvement has been small or negligible among the most deprived groups. As a result, the deprivation gaps in relative survival widened during the 1990s, even after changes in background mortality were adjusted for separately, within each deprivation group.

One-year survival began increasing in the late 1980s, as did the corresponding deprivation gap. A similar pattern is seen for 5-year survival only from the mid-1990s. Long-term survival had not changed until recently, but it is predicted to rise for patients diagnosed during 2000–2001. This pattern may reflect the increasing influence of autologous bone marrow graft and stem cell transplantion, important therapeutic advances introduced from the late 1980s and early 1990s (Richardson *et al*, 2004). The use of drugs such as thalidomide is too recent to have had any effect on survival figures in this study.

In 1986–1990, both incidence and survival were similar in all deprivation groups. The more marked increase in both incidence and survival among the more affluent groups may suggest that these groups have taken more advantage of earlier diagnosis. Early diagnosis has considerable prognostic significance, as autograft is more successful if done at an early stage.

## Figures and Tables

**Table 1:** Trends in relative survival (%) by sex, time since diagnosis and calendar period of diagnosis: England and Wales, adults (15–99 years) diagnosed during 1986–1999 and followed up to 2001

		**Calendar period of diagnosis^a^**						**Average change (%)**		**Prediction^c^ for patients**	
		**1986–1990**		**1991–1995**		**1996–1999**		**every 5 years^b^**		**diagnosed during 2000–2001**	
**Time since diagnosis**		**Survival (%)**	**95% CI**	**Survival (%)**	**95% CI**	**Survival (%)**	**95% CI**	**Survival (%)**	**95% CI**	**Survival (%)**	**95% CI**
1 year	Men	**57.5**	(56.1, 58.9)	**60.6**	(59.3, 61.9)	**62.9**	(61.5, 64.3)	**3.8** ^**^	(1.2, 6.5)	**62.7**	(60.8, 64.7)
	Women	**57.3**	(55.8, 58.7)	**58.5**	(57.2, 59.9)	**60.9**	(59.4, 62.4)	**0.7**	(−2.1, 3.5)	**60.5**	(58.4, 62.6)
5 years	Men	**20.8**	(19.6, 22.1)	**21.6**	(20.5, 22.8)	**25.6**	(23.8, 27.4)	**4.4** ^**^	(1.6, 7.3)	**25.8**	(23.9, 27.8)
	Women	**20.6**	(19.4, 21.9)	**20.4**	(19.3, 21.6)	**23.8**	(22.0, 25.6)	**5.8** ^**^	(3.0, 8.7)	**24.3**	(22.3, 26.3)
10 years	Men	**9.9**	(8.9, 11.0)	**10.7**	(9.5, 12.0)			**0.7**	(−3.5, 4.9)	**14.6**	(12.8, 16.6)
	Women	**9.6**	(8.6, 10.6)	**9.5**	(8.3, 10.7)			**3.5**	(−0.4, 7.3)	**11.7**	(10.0, 13.6)

CI=confidence interval.

a Survival estimated with cohort or complete approach (see Rachet *et al*, 2008).

b Mean absolute change (%) in survival every 5 years, adjusted for deprivation (see Rachet *et al*, 2008).

c Survival estimated with hybrid approach (see Rachet *et al*, 2008).

^**^P<0.01.

**Table 2:** Trends in the deprivation gap in relative survival (%) by sex, time since diagnosis and calendar period of diagnosis: England and Wales, adults (15–99 years) diagnosed during 1986–1999 and followed up to 2001

		**Calendar period of diagnosis^a^**						**Average change (%)**		**Prediction^c^ for patients**	
		**1986–1990**		**1991–1995**		**1996–1999**		**every 5 years^b^**		**diagnosed during 2000–2001**	
**Time since diagnosis**		**Deprivation gap (%)**	**95% CI**	**Deprivation gap (%)**	**95% CI**	**Deprivation gap (%)**	**95% CI**	**Deprivation gap (%)**	**95% CI**	**Deprivation gap (%)**	**95% CI**
1 year	Men	−5.3*	(−9.3, −1.2)	−5.1**	(−8.8, −1.3)	−7.6**	(−11.5, −3.6)	**−1.2**	(−4.2, 1.8)	−7.1*	(−12.7, −1.5)
	Women	**−2.4**	(−6.6, 1.8)	**−2.4**	(−6.3, 1.5)	**0.4**	(−3.9, 4.7)	**1.4**	(−1.7, 4.6)	**0.2**	(−5.9, 6.4)
5 years	Men	**0.4**	(−3.2, 4.1)	**−3.1**	(−6.6, 0.4)	**−4.8**	(−10.0, 0.4)	**−2.8**	(−6.1, 0.4)	−6.7*	(−12.3, −1.1)
	Women	**3.4**	(−0.3, 7.0)	**−1.6**	(−5.1, 1.8)	−7.7**	(−12.9, −2.6)	−5.6**	(−8.8, −2.4)	**−5.8**	(−11.7, 0.1)
10 years	Men	**1.2**	(−1.8, 4.2)	**1.3**	(−2.3, 4.9)			**0.1**	(−4.6, 4.8)	**−4.1**	(−9.5, 1.3)
	Women	4.6**	(1.9, 7.4)	**0.4**	(−3.1, 3.9)			**−4.3**	(−8.7, 0.2)	**−2.7**	(−7.7, 2.3)

CI=confidence interval.

a Survival estimated with cohort or complete approach (see Rachet *et al*, 2008).

b Mean absolute change (%) in the deprivation gap in survival every 5 years, adjusted for the underlying trend in survival (see Rachet *et al*, 2008).

c Survival estimated with hybrid approach (see Rachet *et al*, 2008).

^*^P<0.05; ^**^P<0.01.

## References

[bib1] Berrino F, Estève J, Coleman MP (1995) Basic issues in the estimation and comparison of cancer patient survival. In Survival of Cancer Patients in Europe: the EUROCARE Study. (IARC Scientific Publications No. 132), Berrino F, Sant M, Verdecchia A, Capocaccia R, Hakulinen T, Estève J (eds), pp 1–14. International Agency for Research on Cancer: Lyon

[bib2] Blair A, Kazerouni N (1997) Reactive chemicals and cancer. Cancer Causes Control 8: 473–490 9498905 10.1023/a:1018417623867

[bib3] Brenner H, Rachet B (2004) Hybrid analysis for up-to-date long-term survival rates in cancer registries with delayed recording of incident cases. Eur J Cancer 40: 2494–2501 15519525 10.1016/j.ejca.2004.07.022

[bib4] Coleman MP, Estève J, Damiecki P, Arslan A, Renard H (1993) Trends in Cancer Incidence and Mortality (IARC Scientific Publications No. 121). International Agency for Research on Cancer: Lyon 10.3109/9780415874984-2 8258476

[bib5] IARC (2000) IARC Monographs on the Evaluation of Carcinogenic Risks to Humans. Volume 75. Ionizing Radiation, Part I: X- and Gamma (y) Radiation and Neutrons. International Agency for Research on Cancer: Lyon

[bib6] Lynge E, Anttila A, Hemminki K (1997) Organic solvents and cancer. Cancer Causes Control 8: 406–419 9498902 10.1023/a:1018461406120

[bib7] Quinn MJ, Babb P, Brock A, Kirby L, Jones J (2001) Cancer Trends in England and Wales 1950–1999. Studies on Medical and Population Subjects No. 66. Office for National Statistics: London

[bib8] Rachet B, Woods LM, Mitry E, Riga M, Cooper N, Quinn MJ, Steward J, Brenner H, Estève J, Sullivan R, Coleman MP (2008) Cancer survival in England and Wales at the end of the 20th century. Br J Cancer 99(Suppl 1): S2–S10 18813248 10.1038/sj.bjc.6604571 PMC2557545

[bib9] Richardson P, Hideshima T, Anderson KC (2004) Multiple myeloma and related disorders. In Clinical Oncology. Abeloff MD, Armitage JO, Niederhuber JE, Kastan MB, McKenna WG (eds) (3rd edn), pp 2955–2984. Elsevier: Philadephia

[bib10] Stellman JM (1998) Encyclopaedia of Occupational Health and Safety. 4th edn. International Labour Office: Geneva

